# Infantile Diarrhea, with Special Reference to the Use of “Lactated Milk”

**Published:** 1907-07

**Authors:** 


### INFANTILE DIARRHEA, WITH SPECIAL REFERENCE TO THE USE OF "LACTATED MILK."

Batten says that the preparation of lactated milk is a matter of no great difficulty, but requires a good deal of care and attention to detail.

Fresh milk is obtained. This is boiled in a saucepan or, what the author has found most convenient for a small quantity, a "Gourmet milk sterilizer" in order to sterilize it. The boiled milk is now decanted into a sterilized bottle and the stopper closed. The milk is then stood in cold water until the temperature has fallen to 96 degrees F. To every 11 ounces of milk one tube (about 3 drachms) of the fluid "lactobacilline" (lactone tablets in U. S.) is then added, and in order to mix it with the milk the bottle is well shaken. The bottle is stood in a water-bath or incubtor at the temperature of 96 degree F. for seven hours; during this time the milk becomes sour, and a clot more or less firm is formed in the milk. At the end of this period the milk is like a feebly-set junket. The bottle is now removed from the bath and is placed in the ice chest, and here it remains for twelve hours; during this period the clot undergoes some solution, and at the end of the period a creamy fluid is obtained which is sour in taste but is quite agreeble to drink, and most children will take it readily when diluted with water and a small quantity of sugar added. According to the preparers of the "lactobaccilline" this dissolution of the clot should not take place, and the milk which is prepared by them remains in a solid condition, but can be broken up with a spoon and can be drunk out of a tumbler.

The writer has thought it not improbable that the liquefaction of the clot formed was due to the action of a "liquefying" lactic acid bacillus which had a low optimum temperature.

Whatever was the cause of the liquefaction of the clot it did not impair the efficiency or taste of the milk, and it occurred with such regularity that the author was of the opinion that it must be due to something contained in the liquid "lactobaccilline."

He has had failures in the preparation of the milk; on one occasion the milk became bitter, due apparently to butyric fermentation, and on another gas was formed from the presence of yeast. On another the milk would not clot. He has endeavored to carry on the process of lactic acid fermentation from one specimen of prepared milk to another. This is not attended with success, chiefly because it is impossible to completely sterilize the milk in the first instance by boiling, and during the process of lactic acid fermentation the spores of other organisms developed, and develop actively in the second milk inoculated.

The question will rightly be asked, What is the organism which gives rise to the formation of lactic acid in milk? There are numerous organisms of different types which have the power of producing lactic acid in milk; some of these are harmless, others are pathogenic. In the preparation of "lactobaccilline" two organisms are present: (1) a long rod baccillus growing in chains, Gram-staining, and known as the bacillus acidi lactici, Bulgarian variety; (2) a short rod, or streptococcus acidi lactici, European variety.

The first is the most active in formation of lactic acid, but if allowed to act alone gives rise to a too acid taste to the milk.—Clinical Jour., Dec. 19th, 1906.

